# Reduced rate of MDROs after introducing ‘water-free patient care’ on a large intensive care unit in the Netherlands

**DOI:** 10.1186/2047-2994-4-S1-O40

**Published:** 2015-06-16

**Authors:** J Hopman, R Bos, A Voss, E Kolwijck, P Sturm, P Pickkers, A Tostmann, HVD Hoeven

**Affiliations:** 1 Medical Microbiology/Unit Infection Control, Radboud University Medical Center, Nijmegen, Netherlands; 2 Medical Microbiology, Radboud University Medical Center, Nijmegen, Netherlands; 3 Medical Microbiology, Laurentius Ziekenhuis, Roermond, Netherlands; 4 Intensive Care, Radboud University Medical Center, Nijmegen, Netherlands

## Introduction

Environmental contamination of the patient surroundings is considered of importance in acquiring hospital-associated infections. Sinks and the proximity of water in the patient zone are associated with outbreaks.

## Objectives

To evaluate whether multi-drug resistant organism (MDRO) colonization was reduced after removal of sinks from the intensive care unit (ICU) patient rooms.

## Methods

In the summer of 2014, sinks were removed from all patient rooms at all intensive care units and a water-free method of patient care was introduced.

We conducted a retrospective clinical intervention study and included all patients who were admitted to the ICU during a 6-month pre-intervention and a 6-month post intervention period. We analysed microbiological data of cultures that were collected during the study period. The main outcome of this study was MDR Gram-negative bacteria colonization. These rates were calculated as the number of positive culture results for each pathogen per 1000 ICU admission days.

## Results

During the pre-intervention period, 815 patients were admitted to the ICU, with a total of 3603 admission days. In the post-intervention period, 762 patients were admitted to the ICU, accounting for 3386 admission days. On admission to the ICU, the overall colonisation rate with Gram-negative rods in the pre-intervention period was similar to the post-intervention period. However, when limiting the analysis to positive results of cultures collected at least 2, 5, 7, 10 or 14 days after admission, a large statistically significant difference was demonstrated between the pre and post intervention period. When focussing on the typical hospital pathogens (including MDROs) the difference between pre- and post-intervention was even more apparent, with a rate ratio of 0.44 (95% CI 0.22-0.86; P=0.01).

## Conclusion

The removal of sinks from the patient rooms and the introduction of ‘water-free patient care’ resulted in a significant reduction of colonization with MDR Gram-negative bacteria. The effect on colonization is most evident in patients admitted for longer periods at the ICU.

## Disclosure of interest

None declared.

